# The Annual American Men’s Internet Survey of Behaviors of Men Who Have Sex With Men in the United States: Key Indicators Report 2018

**DOI:** 10.2196/21812

**Published:** 2021-03-04

**Authors:** Sarah Wiatrek, Maria Zlotorzynska, Ramona Rai, Patrick Sullivan, Travis Sanchez

**Affiliations:** 1 Rollins School of Public Health Emory University Atlanta, GA United States

**Keywords:** HIV, internet, men who have sex with men, sexually transmitted infections, surveillance, survey

## Abstract

The American Men’s Internet Survey (AMIS) is an annual web-based behavioral survey conducted in the United States of men who have sex with men (MSM). This rapid surveillance report describes the sixth cycle of data collection (September-December 2018; AMIS 2018). The key indicators were the same as those previously reported for past AMIS cycles. The AMIS methodology has not substantively changed since AMIS 2017. MSM were recruited from a variety of websites using banner advertisements and email blasts. In addition, participants from AMIS 2017 who agreed to be recontacted for future research were emailed a link to AMIS 2018. Men were eligible to participate if they were aged ≥15 years, resided in the United States, provided a valid US ZIP code, and reported ever having sex with a man or identified as gay or bisexual. The analysis was limited to those who reported having oral or anal sex with a male partner in the past 12 months. We examined demographic and recruitment characteristics using multivariable regression modeling (*P*<.05) stratified by the participants’ self-reported HIV status. The AMIS 2018 round of data collection resulted in 10,129 completed surveys from MSM representing every US state, Puerto Rico, and Guam. Most participants were non-Hispanic White, aged between 15 and 24 years, living in urban areas in the southern United States, and recruited from general social networking websites. The plurality (4230/10,129, 41.76%) of participants was in the youngest age group, 15-24 years, followed by the 40 years and older age group (3088/10,129, 30.49%). The self-reported HIV prevalence was 6.08% (616/10,129). Compared with HIV-negative or unknown status participants, HIV-positive participants were more likely to have had anal sex without a condom with a male partner in the past 12 months (adjusted odds ratio [aOR] 2.02, 95% CI 1.63-2.50) and more likely to have had anal sex without a condom with a serodiscordant or an unknown status partner (aOR 3.90, 95% CI 3.27-4.66). The reported use of marijuana in the past 12 months was higher among HIV-positive participants than among HIV-negative or unknown status participants (aOR 1.39, 95% CI 1.15-1.68). The reported use of methamphetamines and other illicit substances in the past 12 months was higher among HIV-positive participants than among HIV-negative or unknown status participants (aOR 3.42, 95% CI 2.41-4.87 and aOR 1.90, 95% CI 1.56-2.32, respectively). Most HIV-negative or unknown status participants (6838/9513, 71.88%) reported ever taking an HIV test previously, and 52.51% (4995/9513) of the participants reported undergoing HIV testing in the past 12 months. HIV-positive participants were more likely to report testing and diagnosis of sexually transmitted infections than HIV-negative or unknown status participants (aOR 3.50, 95% CI 2.89-4.24 and aOR 2.61, 95% CI 2.10-3.25, respectively).

## Introduction

The American Men’s Internet Survey (AMIS) is an annual web-based behavioral survey conducted in the United States of men who have sex with men (MSM). AMIS was developed to produce timely data from large-scale monitoring of behavior trends among MSM recruited on the web. It was designed to complement the Centers for Disease Control and Prevention’s National HIV Behavioral Surveillance (NHBS) system, which collects data on MSM in major US cities every 3 years through venue-based recruitment [1]. An increasing number of MSM are meeting sexual partners through the internet and may have different patterns of sexual risk and HIV testing behaviors compared with MSM recruited through physical venues. AMIS is able to generate annual snapshots of behaviors in a large sample of internet-using MSM with broad geographic diversity as a supplement to venue-based studies, such as the NHBS system. We were also able to collect, update, and share state-level data with public health authorities to inform issues of local relevance by using AMIS.

The methods and past AMIS cycle data (AMIS 2013, AMIS 2014, AMIS 2015, AMIS 2016, and AMIS 2017) have been previously published [2-6].

This supplemental report has updated the existing information with the data collected in AMIS 2018. The methods in AMIS 2018 have not changed from the previously published methods, unless otherwise noted. An in-depth analysis, discussion, and limitations of multiyear trends for indicators reported herein have been published and include data for the first 4 cycles of AMIS (AMIS 2013 to AMIS 2016) [7].

## Methods

### Recruitment and Enrollment

Similar to the previous year’s recruitment process, AMIS participants were recruited through convenience sampling from a variety of websites using banner advertisements or email blasts to members of the website (hereafter referred to generically as *ads*). For AMIS 2018, data were collected from September 2018 to December 2018. The survey was not incentivized. Men who clicked on the ads were taken directly to the survey website hosted on a secure server administered by SurveyGizmo. Recruitment was also done by emailing participants from the previous cycle of AMIS (AMIS 2017) who consented to be recontacted for future studies. To be eligible for the survey, participants had to be aged ≥15 years, be cisgender male, reside in the United States, and report that they either had oral or anal sex with a male partner at least once in the past or identify as gay or bisexual (hereafter referred to as MSM). Persons who were aged <15 years or refused to provide their age were not asked any other screening questions. MSM who met the eligibility criteria and consented to participate in the study started the web-based survey immediately. The full questionnaire for AMIS 2018 is presented in Multimedia Appendix 1.

Several data cleaning steps were performed on the raw data set of eligible responses to obtain the final analysis data set, in the same manner as in previous AMIS cycles [2-6]. Briefly, these steps were as follows: deduplication; limiting to surveys deemed successful, that is, observations with no missing values for the first question of at least two consecutive sections; limiting to participants who reported having oral or anal sex with a male partner in the past 12 months; and ZIP code validation. These steps are further described in detail.

First, to deduplicate survey responses, demographic data for near-complete (>70%) survey responses with nonunique internet protocol addresses were compared, and responses that showed a 100% match for age, race, ethnicity, ever having sex with a woman, and email address were considered to be duplicate responses. Only the observation with the highest survey completion was retained. The data set was, then, limited to those surveys that were deemed successful. Finally, the data set was restricted to include participants who reported having oral or anal sex in the past 12 months and who provided a valid US ZIP code. ZIP codes were validated in the same manner as done in AMIS 2017 [6]. Valid US ZIP codes were those that could be matched to the ZIP code of county crosswalk files created by the US Department of Housing and Urban Development [8]. Any ZIP codes that could not be matched to this list were then hand validated by checking against the ZIP code locator tool on the US Postal Service website [9]. ZIP codes that could not be found were classified as invalid.

### Human Subjects Protections

The study was conducted in compliance with federal regulations governing the protection of human subjects and was reviewed and approved by our institution’s human subjects research review board. No incentive was provided to the participants. Data sets for analyses were stored on secure data servers with access only granted to study staff. The study data are protected under a federal certificate of confidentiality that prevents legal action to force data release.

### Measures and Analyses

For the AMIS 2018 analyses, participants were categorized as either AMIS 2017 participants who took the survey again or new participants from the website or app based on the target audience and purpose: gay social networking (n=2), gay general interest (n=1), general social networking (n=4), and geospatial social networking (n=2). Recruitment outcomes and demographic characteristics for the AMIS 2018 participants are presented in the first two tables, and thereafter, they are recategorized to how they were originally recruited in AMIS 2017. We did not provide the names of the websites or apps to preserve operator and client privacy, particularly when a category has only one operator. Participants whose data were eligible, unduplicated, and successful and who provided consent; reported having male-male sex in the past 12 months; and provided a valid US ZIP code were included in analyses of participant characteristics and behavior.

To facilitate comparisons, the key indicators and analytic approach used in AMIS were designed to mirror those used by the NHBS system [10]. Population density was defined in the same manner as defined in AMIS 2017 and was based on the National Center for Health Statistics Rural-Urban classification scheme for counties [11]. The self-reported HIV status was categorized as HIV-positive, HIV-negative, or unknown, consistent with surveillance reports produced by the NHBS system [10]. In total, 3 substance use behaviors in the past 12 months were assessed: use of nonprescribed marijuana, use of methamphetamines, and use of any illicit drug other than marijuana or methamphetamines. All other indicators assessed remained unchanged from AMIS 2017 [6].

The analysis methods for AMIS 2018 did not substantively differ from those previously published but are repeated in this report for clarity. Overall, chi-square tests were used to identify whether participant characteristics differed significantly among recruitment sources. Multivariable logistic regression modeling was used to determine significant differences in behaviors based on the self-reported HIV status while controlling for race or ethnicity, age group, NHBS city residency, and type of recruitment website. The metropolitan statistical areas included in the NHBS system in 2018 were as follows: Atlanta, Georgia; Boston, Massachusetts; Chicago, Illinois; Dallas, Texas; Denver, Colorado; Detroit, Michigan; Houston, Texas; Los Angeles, California; Memphis, Tennessee; Miami, Florida; Nassau-Suffolk, New York; New Orleans, Louisiana, New York City, New York; Newark, New Jersey; Philadelphia, Philadelphia; Portland, Oregon; San Diego, California; San Francisco, California; San Juan, Puerto Rico; Seattle, Washington; Virginia Beach-Norfolk, Virginia; and Washington, District of Columbia. HIV testing behaviors were only examined among those who did not report that they were living with HIV, and these data were presented in participant characteristics. The multivariable logistic regression results were presented as adjusted odds ratios (aORs) with 95% CI and Wald chi-square *P* values to denote an independently significant difference in the behavior for each subgroup compared with a reference group. Statistical significance was set at *P*=.05.

## Results

### Recruitment Outcomes

AMIS 2018 was conducted from September 2018 to December 2018 and resulted in 91,142 persons screened for eligibility (Table 1). Of the 3713 participants who completed the AMIS 2017 survey and were emailed links to the AMIS 2018 survey, 39.94% (1483/3713) completed the screening. Almost half (42,011/91,142, 46.09%) of the participants who completed the screening process were eligible to participate. The most common reason for ineligibility was not ever having male-male sex or not identifying as gay or bisexual. Almost all (40,847/42,011, 97.23%) of the participants who were eligible consented to participate in the survey. A total of 6595 (6595/40,847, 16.15%) surveys were likely from duplicate participants. Among unduplicated surveys, 35.75% (12,246/34,252) were considered successful. Most successful surveys were from men who reported having sex with another male in the past 12 months (10,232 /12,246, 83.55%). Almost all of these surveys (10,129/10,232, 98.99%) provided a valid US ZIP code. Overall, the completion rate was 11.1%, with an analytical sample consisting of 10,129 surveys from 91,142 screened participants. The median survey completion time, including eligibility screening, was 20.7 minutes (IQR=16.5-27.4).

**Table 1:** Recruitment outcomes for the American Men’s Internet Survey, United States, 2018.

Recruitment outcomes			Total		Gay social networking (n=2)^a^		General gay interest (n=1)^a^		General social networking (n=4)^a^		Geospatial social networking (n=2)^a^		AMIS^b^ 2017 participants
Screened^c^, N			91,142		1288		197		80,768		7406		1483
**Ineligible^d^, n (%)**			49,131 (53.91)		279 (21.66)		140 (71.07)		46,689 (57.81)		1857 (25.07)		166 (11.19)
	Not >15 years of age^e^	22,659 (46.12)		115 (41.22)		13 (9.29)		21,507 (46.06)		951 (51.21)		73 (43.98)	
	Not male^e^	38,832 (79.04)		226 (81)		48 (34.29)		36,830 (78.88)		1590 (85.62)		138 (83.13)	
	Not MSM^e,f^	48,398 (98.51)		257 (92.11)		53 (37.86)		46,233 (99.02)		1697 (91.38)		158 (95.18)	
	Nonresident^e^	36,714 (74.73)		220 (78.85)		131 (93.57)		34,637 (74.19)		1597 (86)		129 (77.71)	
Eligible^c^, n (%)			42,011 (46.09)		1009 (78.34)		57 (28.93)		34,079 (42.19)		5549 (74.93)		1317 (88.81)
Consented^g^, n (%)			40,847 (97.23)		964 (95.54)		55 (96.49)		33,087 (97.09)		5429 (97.84)		1312 (99.62)
Unduplicated^h^, n (%)			34,252 (83.85)		859 (89.11)		51 (92.73)		27,527 (83.20)		4639 (85.45)		1176 (89.63)
Success^i^, n (%)			12,246 (35.75)		489 (56.93)		39 (76.47)		8150 (29.61)		2537 (54.69)		1031 (87.67)
MSM in the past 12 months^j^, n (%)			10,232 (83.55)		434 (88.75)		32 (82.05)		6424 (78.82)		2395 (94.40)		947 (91.85)
Valid ZIP code^k^, n (%)			10,129 (98.99)		430 (99.08)		32 (100)		6351 (98.86)		2375 (99.16)		941 (99.37)

^a^Refers to the number of websites or apps in this category.

^b^AMIS: American Men’s Internet Survey.

^c^Proportion of participants who started the screening questionnaire.

^d^Proportion of total participants screened. Participants who did not complete the screening questionnaire were considered ineligible.

^e^Proportion of ineligible participants, including those who did not respond to the question.

^f^MSM: men who have sex with men or identify as gay or bisexual.

^g^Proportion of eligible participants.

^h^Proportion of participants who consented. Deduplication removes participants who were marked as duplicates using the internet protocol address and demographic data matching.

^i^Proportion of unduplicated participants. Success removes participants who do not pass the test for completeness.

^j^Proportion of successes.

^k^Proportion of men who had sex with men in the past 12 months. Valid US ZIP codes were those that could be matched to the ZIP code for county crosswalk files created by the US Department of Housing and Urban Development. Any ZIP codes that could not be matched to this list were then hand validated by checking against the ZIP code locator tool on the US Postal Service website. ZIP codes that could not be found were classified as invalid.

### Participant Characteristics

**Figure 1:**
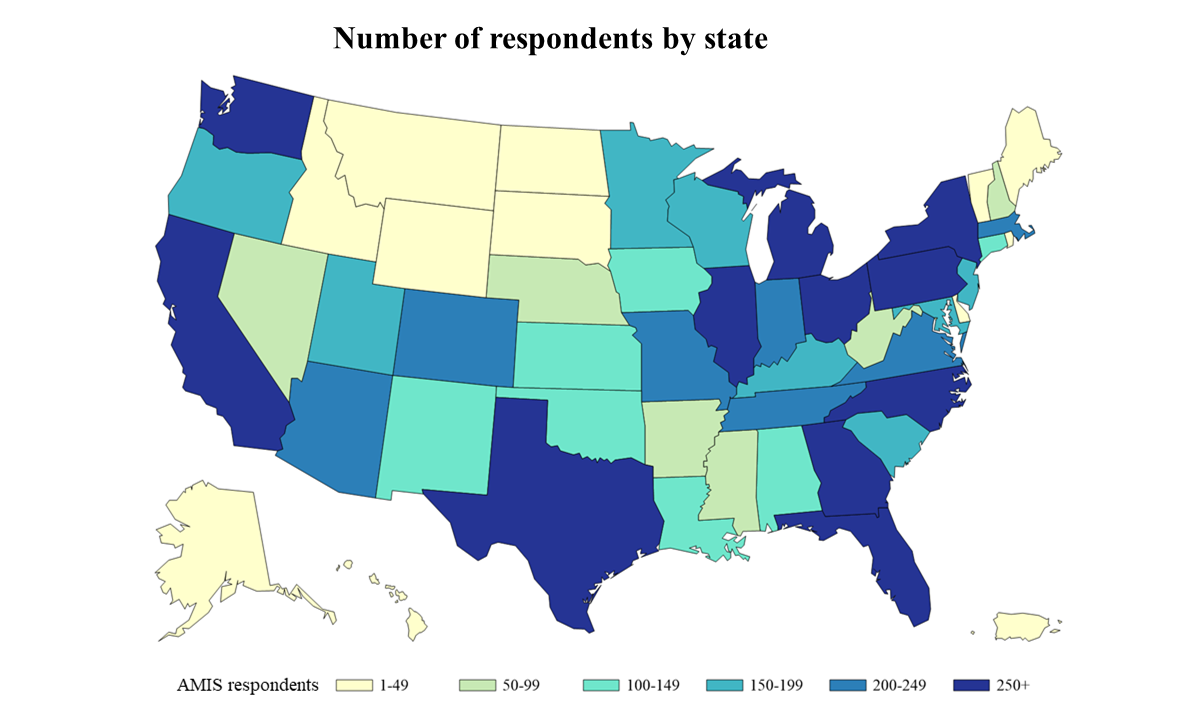
The number of men who have sex with men who participated in the American Men’s Internet Survey (AMIS) by state, 2018.

In total, 69.22% (7011/10,129) of the participants included in this report were non-Hispanic White and 41.76% (4230/10,129) were aged 15-24 years; the most common region of residence was the south, followed by the west (Table 2). Participants were recruited from all US states, and there were at least 100 participants each from 29 states and the District of Columbia (Figure 1). About one-third (3338/10,129, 32.95%) of participants resided in an NHBS city, and about the same proportion (3680/10,129, 36.33%) lived in an urban county. Overall, 6.08% (616/10,129) of participants were living with HIV, 66.39% (6725/10,129) were HIV negative, and 27.52% (2788/10,129) had an unknown HIV status. All participant characteristics differed significantly based on the recruitment source, except NHBS city resident (Table 2).

**Table 2:** Characteristics of men who have sex with men in the American Men’s Internet Survey by recruitment type, United States, 2018.

Participant characteristics			Total		Gay social networking (n=2)^a^		General gay interest (n=1)^a^		General social networking (n=3)^a^		Geospatial social networking (n=2)^a^		AMIS^b^ 2016 participants		*P* value^c^	
**Race or ethnicity, n (%)**																<.001
	Black, non-Hispanic	553 (5.46)		36 (8.4)		<5 (—)^d^		337 (5.3)		127 (5.3)		52 (5.5)				
	Hispanic	1630 (16.09)		32 (7.4)		<5 (—)		1220 (19.21)		269 (11.33)		106 (11.26)				
	White, non-Hispanic	7011 (69.22)		333 (77.4)		24 (75)		4128 (65)		1801 (75.83)		725 (77.05)				
	Other or multiple races	749 (7.39)		16 (3.7)		<5 (—)		546 (8.6)		133 (5.6)		51 (5.4)				
**Age (years), n (%)**																<.001
	15-24	4230 (41.76)		18 (4.19)		5 (15.63)		3919 (61.71)		153 (6.44)		135 (14.35)				
	25-29	1308 (12.91)		19 (4.42)		<5 (—)		968 (15.24)		197 (8.29)		123 (13.07)				
	30-39	1503 (14.84)		63 (14.65)		5 (15.63)		774 (12.19)		484 (20.38)		177 (18.81)				
	≥40	3088 (30.49)		330 (76.74)		21 (65.63)		690 (10.86)		1541 (64.88)		506 (53.77)				
**Region, n (%)**																.02
	Northeast	1632 (16.11)		89 (20.70)		7 (21.88)		1007 (15.86)		351 (14.78)		178 (18.92)				
	Midwest	2198 (21.70)		93 (21.63)		<5 (—)		1397 (22)		509 (21.43)		195 (20.72)				
	South	3865 (38.16)		163 (37.91)		11 (34.38)		2441 (38.43)		906 (38.15)		344 (36.56)				
	West	2426 (23.95)		85 (19.77)		10 (31.25)		1502 (23.65)		606 (25.52)		223 (23.70)				
	US-dependent areas	8 (0.08)		<5 (—)		<5 (—)		<5 (—)		<5 (—)		<5 (—)				
**NHBS^e^ city resident, n (%)**																.12
	Yes	3338 (32.95)		133 (30.93)		15 (46.88)		2022 (31.84)		782 (32.93)		386 (41.02)				
	No	6791 (67.05)		297 (69.07)		17 (53.13)		4329 (68.16)		1593 (67.07)		555 (58.98)				
**Population density^f^, n (%)**																<.001
	Urban	3680 (36.33)		129 (30)		17 (53.13)		2187 (34.44)		906 (38.15)		441 (46.87)				
	Suburban	2110 (20.83)		112 (26.05)		5 (15.63)		1351 (21.27)		477 (20.08)		165 (17.53)				
	Small or medium metropolitan	3317 (32.75)		138 (32.09)		6 (18.75)		2181 (34.34)		715 (30.11)		277 (29.44)				
	Rural	1013 (10)		51 (11.86)		<5 (—)		627 (9.87)		274 (11.54)		57 (6.06)				
**Self-reported HIV status, n (%)**																<.001
	Positive	616 (6.08)		42 (9.8)		<5 (—)		205 (3.2)		255 (10.74)		110 (11.7)				
	Negative	6725 (66.39)		300 (69.77)		25 (78.13)		3758 (59.17)		1868 (78.65)		774 (82.25)				
	Unknown	2788 (27.52)		88 (20.47)		<5 (—)		2388 (37.6)		252 (10.61)		57 (6.06)				
Total, n (%)			10,129 (100)		430 (4.26)		32 (0.32)		6351 (62.70)		2375 (23.45)		941 (9.29)		N/A^g^	

^a^Refers to the number of websites or apps in this category.

^b^AMIS: American Men’s Internet Survey.

^c^A chi-square test for the difference in characteristics between recruitment types.

^d^Percentage is not reported due to an insufficient n.

^e^NHBS: National HIV Behavioral Surveillance.

^f^The National Center for Health Statistics urban or rural category could not be assigned to 10 participants living in US territories.

^g^N/A: not applicable.

### Sexual Behaviors

Approximately two-thirds (6926/10,129, 68.37%) of participants reported having anal sex without a condom with another male in the past 12 months, and about one-fifth (2390/10,129, 23.59%) of participants reported doing so with a partner of a discordant or an unknown HIV status (Table 3). Compared with HIV-negative or unknown status participants, those who were living with HIV were significantly more likely to report anal intercourse without a condom (aOR 2.02, 95% CI 1.63-2.50), including with male partners who were of a discordant or an unknown status (aOR 3.90, 95% CI 3.27-4.66). Stratified by the serostatus group, anal intercourse without a condom differed significantly for only HIV-negative or unknown status participants by race or ethnicity, age group, and recruitment website. Anal intercourse without a condom with partners of a discordant or an unknown HIV status differed significantly by race or ethnicity, residence in an NHBS city, and recruitment type for HIV-negative or unknown status participants only.

**Table 3:** Sexual behaviors with male partners of men who have sex with men in the American Men’s Internet Survey, United States, 2018.

Participant characteristics by HIV status				Participants (N)		Sexual behaviors with male partners in the past 12 months							
						Anal intercourse without a condom					Anal intercourse without a condom with a partner of a discordant or an unknown HIV status		
						n (%)		*P* value^a^		n (%)			*P* value^a^
**HIV positive**				616		499 (81)		<.001^b^		338 (54.87)			<.001^b^
	**Race or ethnicity**												
		Black, non-Hispanic	97		69 (71.13)		.31		39 (40.20)			.09	
		Hispanic	73		58 (79.45)		.92		41 (56.16)			. 35	
		White, non-Hispanic	406		341 (83.99)		Ref^c^		240 (59.11)			Ref^c^	
		Other or multiple races	29		21 (72.41)		.40		13 (44.83)			.42	
	**Age (years)**												
		15-24	43		37 (86.05)		.76		24 (55.81)			.86	
		25-29	40		37 (92.50)		.12		24 (60)			.29	
		30-39	104		86 (82.69)		.36		60 (57.69)			>.99	
		≥40	429		339 (79.02)		Ref^c^		230 (53.61)			Ref^c^	
	**NHBS^d^ city resident**												
		Yes	255		203 (79.61)		.89		137 (53.73)			.89	
		No	361		296 (81.99)		Ref^c^		201 (55.68)			Ref^c^	
	**Recruitment type**												
		Gay social networking	59		45 (76.27)		>.99		33 (55.93)			.83	
		General gay interest	7		5 (71.43)		.52		4 (57.14)			.95	
		General social networking	260		205 (78.85)		Ref^c^		128 (49.23)			Ref^c^	
		Geospatial social networking	290		244 (84.14)		.10		173 (59.66)			.43	
**HIV negative or unknown status**				9513		6427 (67.56)		Ref^b,c^		2052 (21.57)			Ref^b,c^
	**Race or ethnicity**												
		Black, non-Hispanic	456		294 (64.47)		.32		130 (28.51)			.008	
		Hispanic	1557		1049 (67.37)		.05		367 (23.57)			.79	
		White, non-Hispanic	6605		4526 (68.52)		Ref^c^		1357 (20.55)			Ref^c^	
		Other or multiple races	720		449 (62.36)		.03		166 (23.05)			.78	
	**Age (years)**												
		15-24	4187		2626 (62.72)		<.001		799 (19.08)			.08	
		25-29	1268		965 (76.10)		<.001		292 (23.03)			.08	
		30-39	1399		1072 (76.63)		<.001		308 (22.02)			.37	
		≥40	2659		1764 (66.34)		Ref^c^		653 (24.56)			Ref^c^	
	**NHBS city resident**												
		Yes	3083		2103 (68.21)		.41		732 (23.74)			.002	
		No	6430		4324 (67.25)		Ref^c^		1320 (20.53)			Ref^c^	
	**Recruitment type**												
		Gay social networking	503		280 (55.66)		<.001		108 (21.47)			.73	
		General gay interest	42		25 (59.52)		.57		6 (14.29)			.23	
		General social networking	6587		4385 (66.57)		Ref^c^		1266 (19.22)			Ref^c^	
		Geospatial social networking	2366		1725 (72.91)		<.001		668 (28.23)			<.001	

^a^Wald chi-square *P* values from the multivariate logistic regression model comparing behavior (yes vs no) between groups with specific characteristics and a reference group.

^b^Wald chi-square *P* values from the multivariate logistic regression model comparing behavior (yes vs no) among HIV-positive participants and HIV-negative or unknown serostatus participants. Model controlled for race or ethnicity, age, NHBS system city residency, and recruitment type.

^c^Ref: The reference group being compared to within the multivariate logistic regression models.

^d^NHBS: National HIV Behavioral Surveillance.

### Substance Use Behaviors

In total, 31.93% (3235/10,129) of participants reported using marijuana, 2.31% (234/10,129) reported using methamphetamines, and 20.43% (2069/10,129) reported using other illicit substances in the past 12 months (Table 4). Compared with participants with HIV negative or unknown status, HIV-positive participants were significantly more likely to report the use of marijuana (aOR 1.39, 95% CI 1.15-1.68), methamphetamines (aOR 3.42, 95% CI 2.41-4.87), and other illicit substances (aOR 1.90, 95% CI 1.56-2.32) in the past 12 months. Among HIV-positive participants, the use of marijuana varied significantly by age, and the use of other illicit substances varied significantly by race or ethnicity. In addition, the use of marijuana, methamphetamines, and other illicit substances differed significantly by age and race or ethnicity among HIV-negative or unknown status participants. In this group, the use of marijuana and other illicit substances differed significantly by residence in an NHBS city, and the use of marijuana differed significantly by the recruitment website.

**Table 4:** Substance use behaviors of men who have sex with men in the American Men’s Internet Survey, United States, 2018.

Participant characteristics by HIV status				Participants (N)		Used marijuana					Used methamphetamines					Used other substances		
						n (%)		*P* value^a^		n (%)			*P* value^a^		n (%)			*P* value^a^
**HIV positive**				616		189 (30.68)		.001^b^		50 (8.12)			<.001^b^		169 (27.44)			<.001^b^
	**Race or ethnicity**																	
		Black, non-Hispanic	97		18 (18.56)		.12		<5 (—)^c^			.21		8 (8.24)			<.001	
		Hispanic	73		22 (30.14)		.91		5 (6.85)			.85		21 (28.77)			.15	
		White, non-Hispanic	406		137 (33.74)		Ref^d^		37 (9.11)			Ref^d^		130 (32.02)			Ref^d^	
		Other or multiple races	29		8 (27.58)		.81		<5 (—)			.95		7 (24.14)			.77	
	**Age (years)**																	
		15-24	43		22 (51.16)		.002		<5 (—)			.81		12 (27.91)			.82	
		25-29	40		14 (35)		.88		<5 (—)			.24		13 (32.50)			.62	
		30-39	104		42 (40.38)		.96		11 (10.58)			.19		36 (34.62)			.20	
		≥40	429		111 (25.87)		Ref^d^		34 (7.93)			Ref^d^		108 (25.17)			Ref^d^	
	**NHBS^e^ city resident**																	
		Yes	255		81 (31.76)		.22		24 (9.41)			.19		70 (27.45)			.46	
		No	361		108 (29.92)		Ref^d^		26 (7.20)			Ref^d^		99 (27.42)			Ref^d^	
	**Recruitment type**																	
		Gay social networking	59		15 (25.42)		.63		<5 (—)			.97		16 (27.12)			.84	
		General gay interest	7		<5 (—)		.42		<5 (—)			.96		<5 (—)			.37	
		General social networking	260		73 (28.08)		Ref^d^		22 (8.97)			Ref^d^		61 (23.46)			Ref^d^	
		Geospatial social networking	290		100 (34.48)		.06		26 (8.9)			.96		89 (30.69)			.93	
**HIV negative or unknown status**				9513		3046 (32.02)		Ref^b,d^		184 (1.93)			Ref^b,d^		1900 (19.97)			Ref^b,d^
	**Race or ethnicity**																	
		Black, non-Hispanic	456		122 (26.75)		.002		6 (1.32)			.18		60 (13.16)			<.001	
		Hispanic	1557		544 (34.94)		.86		42 (2.70)			.002		337 (21.64)			.10	
		White, non-Hispanic	6605		2071 (31.36)		Ref^d^		117 (1.77)			Ref^d^		1301 (19.70)			Ref^d^	
		Other or multiple races	720		269 (37.36)		.02		13 (1.81)			.81		173 (24.03)			<.001	
	**Age (years)**																	
		15-24	4187		1611 (38.48)		<.001		47 (1.12)			.009		861 (20.56)			.18	
		25-29	1268		427 (33.68)		.01		22 (1.74)			.70		310 (24.45)			<.001	
		30-39	1399		468 (33.45)		.03		41 (2.93)			.01		328 (23.45)			.005	
		≥40	2659		540 (20.31)		Ref^d^		74 (2.78)			Ref^d^		401 (15.08)			Ref^d^	
	**NHBS city resident**																	
		Yes	3083		1100 (35.68)		<.001		57 (1.85)			.35		731 (23.71)			<.001	
		No	6430		1946 (30.26)		Ref^d^		127 (1.98)			Ref^d^		1169 (18.18)			Ref^d^	
	**Recruitment type**																	
		Gay social networking	503		99 (19.68)		.002		21 (4.17)			.10		80 (15.90)			.33	
		General gay interest	42		19 (45.23)		.003		<5 (—)			.93		11 (26.19)			.18	
		General social networking	6587		2294 (34.83)		Ref^d^		84 (1.28)			Ref^d^		1343 (20.39)			Ref^d^	
		Geospatial social networking	2366		632 (26.71)		.15		78 (3.30)			.65		464 (19.61)			.75	

^a^Wald chi-square *P* values from the multivariate logistic regression model comparing behavior (yes vs no) between groups with specific characteristics and a reference group.

^b^Wald chi-square *P* values from the multivariate logistic regression model comparing behavior (yes vs no) among HIV-positive participants and HIV-negative or unknown serostatus participants. Model controlled for race or ethnicity, age, National HIV Behavioral Surveillance system city residency, and recruitment type.

^c^Percentage is not reported due to an insufficient n.

^d^Ref: The reference group being compared to within the multivariate logistic regression models.

^e^NHBS: National HIV Behavioral Surveillance.

### HIV Testing Behaviors

HIV testing behaviors were examined among participants who were not HIV positive (Table 5). Most participants (6836/9513, 71.85%) were previously tested for HIV infection, and 52.51% (4995/9513) of the participants were tested in the past 12 months. HIV testing behavior, both ever tested and tested in the past 12 months, differed significantly by age, residence in an NHBS city, and type of recruitment website.

**Table 5:** HIV testing behaviors of HIV-negative or unknown status men who have sex with men in the American Men’s Internet Survey, United States, 2018.

Participant characteristics			Participants (N)		HIV testing behaviors						
					Ever tested for HIV				Tested for HIV in the past 12 months		
					n (%)		*P* value^a^		n (%)		*P* value^a^
**Race or ethnicity**											
	Black, non-Hispanic	456		339 (74.34)		.46		256 (56.14)		.50	
	Hispanic	1557		1028 (66.02)		.87		799 (51.32)		.66	
	White, non-Hispanic	6605		4871 (73.75)		Ref^b^		3480 (52.69)		Ref^b^	
	Other or multiple races	720		489 (67.92)		.93		377 (52.36)		.73	
**Age (years)**											
	15-24	4187		2115 (55.01)		<.001		1692 (40.41)		<.001	
	25-29	1268		1057 (83.36)		.11		752 (59.31)		.001	
	30-39	1399		1268 (90.64)		<.001		901 (64.40)		<.001	
	≥40	2659		2398 (90.18)		Ref^b^		1650 (62.05)		Ref^b^	
**NHBS^c^ city resident**											
	Yes	3083		2339 (75.87)		<.001		1797 (58.29)		<.001	
	No	6430		4499 (69.97)		Ref^b^		3198 (49.74)		Ref^b^	
**Recruitment type**											
	Gay social networking	503		414 (82.31)		<.001		261 (51.88)		.03	
	General gay interest	42		38 (90.47)		.22		19 (45.24)		.07	
	General social networking	6587		4243 (64.41)		Ref^b^		3063 (46.50)		Ref^b^	
	Geospatial social networking	2366		2128 (89.94)		.08		1640 (69.32)		<.001	
Total			9513		6838 (71.88)				4995 (52.51)		

^a^Wald chi-square *P* values from the multivariate logistic regression model comparing behavior (yes vs no) between groups with specific characteristics and a reference (Ref) group.

^b^Ref: The reference group being compared to within the multivariate logistic regression models.

^c^NHBS: National HIV Behavioral Surveillance.

### Sexually Transmitted Infection Testing and Diagnosis

In total, 42.59% (4314/10,129) of participants reported sexually transmitted infection (STI) testing in the past 12 months, and 10.08% (1022/10,129) of participants reported a diagnosis of STI in the past 12 months. Compared with HIV-negative or unknown status participants, HIV-positive participants were significantly more likely to report STI testing (aOR 3.50, 95% CI 2.89-4.24) and STI diagnosis (aOR 2.61, 95% CI 2.10-3.25) in the past 12 months (Table 6). The most common STI diagnosis among HIV-positive participants was syphilis (78/616, 12.7%), followed by gonorrhea (64/616, 10.4%) and chlamydia (62/616, 10.1%). Gonorrhea was the most common STI diagnosis among HIV-negative or unknown status participants (542/9513, 5.69%), followed by chlamydia (520/9513, 5.47%) and syphilis (282/9513, 2.96%). STI testing and diagnosed with any STI significantly differed by age, residence in an NHBS city, and recruitment website among HIV-negative or unknown status participants. Among HIV-positive participants, STI testing only significantly differed by residence in an NHBS city.

**Table 6:** Sexually transmitted infection testing and diagnosis of men who have sex with men in the American Men’s Internet Survey, United States, 2018.

Participant characteristics by HIV status				Participants (N)		STI^a^ history in the past 12 months						
						Tested for any STI				Diagnosed with any STI		
						n (%)		*P* value^b^		n (%)		*P* value^b^
**HIV positive**				616		448 (72.7)		<.001^c^		135 (21.9)		<.001^c^
	**Race or ethnicity**											
		Black, non-Hispanic	97		73 (75)		.46		24 (25)		.68	
		Hispanic	73		56 (77)		.89		23 (32)		.19	
		White, non-Hispanic	406		290 (71.4)		Ref^d^		80 (19.7)		Ref^d^	
		Other or multiple races	29		21 (72)		.53		6 (21)		.47	
	**Age (years)**											
		15-24	43		37 (86)		.09		13 (30)		.28	
		25-29	40		30 (75)		.72		11 (28)		.92	
		30-39	104		88 (84.6)		.28		33 (31.7)		.19	
		≥40	429		293 (68.3)		Ref^d^		78 (18.2)		Ref^d^	
	**NHBS^e^ city resident**											
		Yes	255		202 (79.2)		.004		62 (24.3)		.32	
		No	361		246 (68.1)		Ref^d^		73 (20.2)		Ref^d^	
	**Recruitment type**											
		Gay social networking	59		41 (70)		.93		10 (17)		.54	
		General gay interest	7		5 (71)		.99		<5 (—)		.48	
		General social networking	260		184 (70.8)		Ref^d^		56 (21.5)		Ref^d^	
		Geospatial social networking	290		218 (75.2)		.28		67 (23.1)		.84	
**HIV negative or unknown status**				9513		3866 (40.64)		Ref^c,d^		887 (9.32)		Ref^c,d^
	**Race or ethnicity**											
		Black, non-Hispanic	456		192 (42.1)		.90		49 (10.8)		.77	
		Hispanic	1557		642 (41.23)		.41		169 (10.85)		.25	
		White, non-Hispanic	6605		2669 (40.41)		Ref^d^		587 (8.89)		Ref^d^	
		Other or multiple races	720		293 (40.7)		>.99		68 (9.4)		.61	
	**Age (years)**											
		15-24	4187		1419 (33.89)		<.001		330 (7.88)		.09	
		25-29	1268		631 (49.76)		<.001		161 (12.70)		<.001	
		30-39	1399		676 (48.32)		<.001		153 (10.94)		.44	
		≥40	2659		1140 (42.87)		Ref^d^		243 (9.14)		Ref^d^	
	**NHBS^e^ city resident**											
		Yes	3083		1425 (46.22)		<.001		389 (12.62)		<.001	
		No	6430		2441 (37.96)		Ref^d^		498 (7.74)		Ref^d^	
	**Recruitment type**											
		Gay social networking	503		183 (36.4)		.72		41 (8.2)		.96	
		General gay interest	42		12 (29)		.06		<5 (—)^f^		.62	
		General social networking	6587		2476 (37.59)		Ref^d^		545 (8.27)		Ref^d^	
		Geospatial social networking	2366		1188 (50.21)		<.001		296 (12.51)		.02	

^a^STI: sexually transmitted infection (includes chlamydia, gonorrhea, and syphilis).

^b^Wald chi-square *P* values from the multivariate logistic regression model comparing behavior (yes vs no) between groups with specific characteristics and a reference (Ref) group.

^c^Wald chi-square *P* values from the multivariate logistic regression model comparing behavior (yes vs no) among HIV-positive participants and HIV-negative or unknown serostatus participants. Model controlled for race or ethnicity, age, NHBS system city residency, and recruitment type.

^d^Ref: The reference group being compared to within the multivariate logistic regression models.

^e^NHBS: National HIV Behavioral Surveillance.

^f^Percentage is not reported due to an insufficient n.

## Discussion

The sixth round of data collection for AMIS was successfully implemented and resulted in over 10,000 surveys from a diverse sample of internet-using MSM residing in all US states. A majority of eligible and enrolled participants were recruited from general social networking, were White, non-Hispanic, aged between 15 and 24 years, and reported being HIV negative. There were notable differences in key behavioral indicators by self-reported HIV status. Compared with HIV-negative or unknown status participants, HIV-positive participants were more likely to have had anal sex without a condom with a male partner in the past year and more likely to have had anal sex without a condom with a serodiscordant or an unknown status partner. The reported use of marijuana, methamphetamines, and other illicit substances in the past year was higher among HIV-positive participants than among HIV-negative or unknown status participants. HIV-positive participants were also more likely to report testing and diagnosis of STIs than HIV-negative or unknown status participants. When stratified by HIV status, some significant differences in these behavioral indicators by demographics and recruitment websites were also observed.

## Appendix

Multimedia Appendix 1 American Men’s Internet Survey 2018 questionnaire.

## Abbreviations

AMIS: American Men’s Internet Survey
aOR: adjusted odds ratio
MSM: men who have sex with men
NHBS: National HIV Behavioral Surveillance System
STI: sexually transmitted infection

## References

[1] Finlayson T, Le B, Smith A, Bowles K, Cribbin M, Miles I, Oster AM, Martin T, Edwards A, DiNenno E (2011). HIV risk, prevention, and testing behaviors among men who have sex with men--National HIV Behavioral Surveillance System, 21 U.S. cities, United States, 2008. MMWR Surveillence Summaries.

[2] Sanchez TH, Sineath RC, Kahle EM, Tregear SJ, Sullivan PS (2015). The annual American men's internet survey of behaviors of men who have sex with men in the United States: protocol and key indicators report 2013. JMIR Public Health Surveill.

[3] Sanchez T, Zlotorzynska M, Sineath C, Kahle E, Sullivan P (2016). The annual American men's internet survey of behaviors of men who have sex with men in the United States: 2014 key indicators report. JMIR Public Health Surveill.

[4] Zlotorzynska M, Sullivan P, Sanchez T (2017). The annual American men's internet survey of behaviors of men who have sex with men in the United States: 2015 key indicators report.. JMIR Public Health Surveill.

[5] Zlotorzynska M, Sullivan P, Sanchez T (2019). The annual American men's internet survey of behaviors of men who have sex with men in the United States: 2016 key indicators report. JMIR Public Health Surveill.

[6] Zlotorzynska M, Cantu C, Rai R, Sullivan P, Sanchez T (2020). The annual American men's internet survey of behaviors of men who have sex with men in the United States: 2017 key indicators report. JMIR Public Health Surveill.

[7] Sanchez TH, Zlotorzynska M, Sineath RC, Kahle E, Tregear S, Sullivan PS (2018). National trends in sexual behavior, substance use and HIV testing among United States men who have sex with men recruited online, 2013 through 2017. AIDS Behav.

[8] HUD USPS ZIP code crosswalk files.

[9] USPS ZIP code lookup.

[10] Centers for Disease ControlPrevention (2014). Hiv infection risk, prevention, and testing behaviors among men who have sex with men. National HIV Behavioral Surveillance, 20 U.S. Cities.

[11] Ingram DD, Franco SJ (2014). 2013 NCHS Urban-rural classification scheme for counties. Vital Health Stat 2.

