# Supplementary material for: The Annual American Men’s Internet Survey of Behaviors of Men Who Have Sex With Men in the United States: Key Indicators Report 2018
Source: JMIR Public Health Surveill. 2021 Mar 4;7(3):e21812. doi: 10.2196/21812 (PMC7974762; doi:10.2196/21812)
Supplement: Multimedia Appendix 1 [file publichealth_v7i3e21812_app1.docx]

**
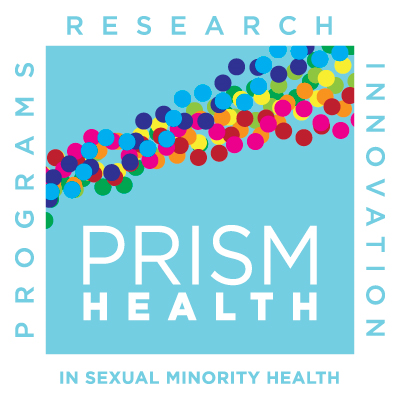
**

**American Men’s Internet Survey (AMIS) 2018:**

Online HIV Behavioral Survey of Men Who Have Sex with Men

*
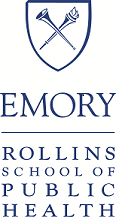
*

**For data requests and additional information:**

**Email**: [amis@emory.edu](mailto:amis@emory.edu)

**Phone**: 404-727-8799

**Address**: PRISM Health

1518 Clifton Road NE

Atlanta, Georgia 30322

# Sex is the Question - 2018


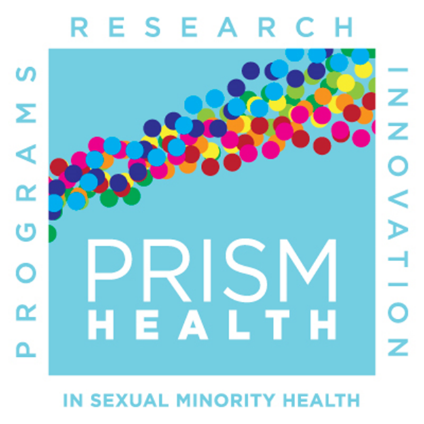


## Eligibility Screener

### Questions marked with * are required.

### How old are you?*

_________________________________________________

## Eligibility screener

#### Since July 2018, have you already completed at least part of Sex is the Question?

( ) No

( ) Yes

( ) I prefer not to answer

( ) Don't know

#### Do you consider yourself to be Hispanic or Latino?*

( ) No

( ) Yes

( ) I prefer not to answer

( ) Don't know

#### Which racial group or groups do you consider yourself to be in? Check all that apply.*

[ ] American Indian or Alaska Native

[ ] Asian

[ ] Black or African American

[ ] Native Hawaiian or Other Pacific Islander

[ ] White

[ ] I prefer not to answer

[ ] Does not apply

[ ] Don't know

#### What country do you live in?*

( ) United States

( ) Mexico

( ) Other country

### What ZIP Code do you live in?*

_________________________________________________

#### How do you describe your current gender identity? You can choose more than one answer.*

[ ] Male

[ ] Female

[ ] Transgender woman (male-to-female transgender)

[ ] Transgender man (female-to-male transgender)

[ ] Other gender identity

[ ] I prefer not to answer

[ ] Don't know

### What is your other gender identity?

_________________________________________________

#### What sex were you assigned at birth?*

( ) Male

( ) Female

( ) Intersex/ambiguous

( ) I prefer not to answer

( ) Don't know

#### Have you ****ever**** had vaginal sex (penis in the vagina) or anal sex (penis in the butt) with a woman?*

( ) No

( ) Yes

( ) I prefer not to answer

( ) Don't know

#### Have you ****ever**** had oral sex (mouth on the penis) with a man?*

( ) No

( ) Yes

( ) I prefer not to answer

( ) Don't know

#### Have you ****ever**** had anal sex (penis in the butt) with a man?*

( ) No

( ) Yes

( ) I prefer not to answer

( ) Don't know

#### Do you consider yourself to be:*

( ) Homosexual, Gay or Lesbian

( ) Heterosexual or Straight

( ) Bisexual

( ) I prefer not to answer

( ) Don't know

#### Do you consider yourself to be:*

( ) Homosexual, Gay or Lesbian

( ) Heterosexual or Straight

( ) Bisexual

( ) Another sexual identity:

( ) I prefer not to answer

( ) Don't know

## Future Contact for Ineligible

#### You are not eligible for this survey, but the PRISM Health team conducts many research projects at Emory University. Would you like to be contacted for potential participation in our future projects?

( ) Yes

( ) No

### Please provide the email address you would like for us to use to contact you for future studies.

_________________________________________________

## Consent

### Thank you for your interest in our survey! The video below will give you more information. Please watch it and indicate below whether you agree to participate.*

( ) I agree to participate in the survey.

( ) I do not agree to participate in the survey.

## Assent

### Thank you for your interest in our survey! The video below will give you more information. Please watch it and indicate below whether you agree to participate.*

( ) I have read the information below. I agree to participate in this survey.

( ) I do not agree to participate in the survey.

## Demographics

#### What is your primary language?

( ) English

( ) Spanish

( ) Another language

( ) I prefer not to answer

#### What other language is your primary language?

_________________________________________________

#### What is the highest level of education you completed?

( ) Never attended school

( ) Less than high school

( ) Some high school

( ) High school diploma or GED

( ) Some college, Associate’s Degree, or Technical Degree

( ) College, post graduate or professional school

( ) I prefer not to answer

( ) Don't know

#### Are you currently enrolled in school?

( ) No

( ) Yes

( ) I prefer not to answer

( ) Don't know

#### What is the highest level of education you completed?

( ) Never attended school

( ) Grades 1 through 8

( ) Grades 9 through 11

( ) Grade 12 or GED

( ) Some college, associate’s degree, or technical degree

( ) Bachelor’s degree

( ) Any post graduate studies

( ) Prefer not to answer

( ) Don’t know

## Demographics: Income

#### What was your household income last year from all sources before taxes? That is, the total amount of money earned ****and shared**** by all people living in your household.

( ) $0 to $19,999 annually ($0 to $1667 monthly)

( ) $20,000 to $39,999 annually ($1668 to $3333 monthly)

( ) $40,000 to $74,999 annually ($3334 to $6250 monthly)

( ) $75,000 or more annually ($6251 or more monthly)

( ) I prefer not to answer

( ) Don't know

### ****Including yourself****, how many people depend on this income?

_________________________________________________

### How many of your dependents are under the age of 18?

_________________________________________________

## Demographics: Hispanic Ethnicity

#### What country were you born in?

#### What state or territory did you live in when you were born?

#### Which of the following describes your Hispanic/Latino heritage? You may choose more than one option.

[ ] Mexican

[ ] Puerto Rican

[ ] Cuban

[ ] Dominican

[ ] Salvadoran

[ ] Guatemalan

[ ] Honduran

[ ] Nicaraguan

[ ] Panamanian

[ ] Costa Rican

[ ] Argentine

[ ] Bolivian

[ ] Brazilian

[ ] Chilean

[ ] Colombian

[ ] Ecuadorian

[ ] Paraguayan

[ ] Peruvian

[ ] Uruguayan

[ ] Venezuelan

[ ] Spanish

[ ] Other: _________

## Demographics: Arrival to US

#### What year did you first come to live in the United States?

#### [TEXT BOX]

#### ( ) Don’t know

### How old were you when you first came to live in the U.S.?

____ years old

#### ( ) Don’t know

#### What are the reason(s) you first came to live in the United States? Check all that apply.

[ ] To improve financial situation (to take a job or find work)

[ ] To live more openly as gay/bi/queer

[ ] To live with or join a lover, boyfriend, or husband

[ ] To be able to marry my male partner

[ ] To study

[ ] To be with family or friends

[ ] I came here as a tourist and decided to stay

[ ] To receive medical care

[ ] To escape violence or persecution for being gay/bi/queer

[ ] To escape violence or persecution for other reasons

[ ] To find political asylum

[ ] It was not my decision to come to the US

[ ] Other reason: _______________

[ ] I prefer not to answer

[ ] Don't know

## Demographics: Housing

#### What best describes the type of housing you ****currently**** live in?

## ( ) Apartment

( ) House (stand-alone unit or condominium)

( ) College dorm or other school-related campus housing

( ) Mobile home/motel/hotel

( ) Single residence occupancy (SRO)/Rooming house/boarding house

( ) Homeless shelter

( ) Other (Please specify): ___________________________

#### ****In the past 12 months,**** did you double up or stay overnight with friends, relatives, or someone you didn’t know well because you didn’t have a regular, adequate, and safe place to stay at night?

( ) No

( ) Yes

( ) I prefer not to answer

( ) Don't know

#### ****In the past 12 months****, were you ever homeless? That is, were you living on the street, in a shelter, in a Single Room Occupancy hotel (SRO), or in a car?

( ) No

( ) Yes

( ) I prefer not to answer

( ) Don't know

## Healthcare

#### What kind of health insurance or health care coverage do you currently have? Choose all that apply.

[ ] A private health plan purchased through an employer

[ ] A private health plan purchased through an exchange (i.e. Obamacare)

[ ] Medicaid or Medicare

[ ] Some other Medical Assistance program

[ ] TRICARE (CHAMPUS)

[ ] Veterans Administration coverage

[ ] Some other health care plan

[ ] I don't currently have any health insurance

[ ] I prefer not to answer

[ ] Don't know

#### ****In the past 12 months****, have you seen a doctor, nurse, or other health care provider about your own health?

( ) No

( ) Yes

( ) I prefer not to answer

( ) Don't know

#### At any of those times you were seen by a doctor or health care provider, were you offered an HIV test? An HIV test checks whether someone has the virus that causes AIDS.

( ) No

( ) Yes

( ) I prefer not to answer

( ) Don't know

#### Did your doctor or health care provider talk to you about sex (gay or straight) or sexual health?

( ) No

( ) Yes

( ) I prefer not to answer

( ) Don't know

## CDC DHAP: Healthcare utilization

#### In the past 12 months, how many times did you see a healthcare provider? Do not include hospitalizations overnight, visits to the emergency room or telephone calls. If you don't quite remember, please answer with your best guess.

( ) 1

( ) 2 to 3

( ) 4 to 5

( ) 6 to 7

( ) 8 to 9

( ) 10 to 12

( ) 13 to 15

( ) 16 or more

( ) I prefer not to answer

( ) Don't know

**In the past 12 months, where did you go to get routine preventative care such as a physical examination or checkup? Please select up to 2 of the most common places.**

[ ] I did not have a regular place or doctor to go to for healthcare

[ ] Private doctor's office

[ ] HIV counseling and testing site

[ ] Public health clinic/community health clinic

[ ] Sexually transmitted disease clinic

[ ] Family planning clinic (like Planned Parenthood)

[ ] Urgent Care or Emergency Room

[ ] Hospital (outpatient department)

[ ] Correctional facility (jail or prison)

[ ] Other (please specify)

## Demographics: Outness

#### Do you consider yourself to be:

( ) Heterosexual or Straight

( ) Homosexual or Gay

( ) Bisexual

( ) Other sexual identity

( ) I prefer not to answer

( ) Don't know

### What is your sexual identity?

_________________________________________________

#### Have you ****ever**** told anyone that you are attracted to or have sex with men?

( ) No

( ) Yes

( ) I prefer not to answer

( ) Don't know

#### Who of the following people have you told that you are attracted to or have sex with men?

|  | **No** | **Yes** | **Does not apply** |
| --- | --- | --- | --- |
| Gay, lesbian, or bisexual friends | ( ) | ( ) | ( ) |
| Friends who are not gay, lesbian, or bisexual | ( ) | ( ) | ( ) |
| Family members | ( ) | ( ) | ( ) |
| Health care provider | ( ) | ( ) | ( ) |
| Employer | ( ) | ( ) | ( ) |
| Fellow employees | ( ) | ( ) | ( ) |

## Substance Use: Alcohol Use

### For the next set of questions, a drink of alcohol is a 12 oz beer, a 5 oz glass of wine, or a 1.5 oz shot of liquor. A 40 oz beer would count as 3 drinks. A cocktail with 2 shots would count as 2 drinks.

#### How often did you have a drink containing alcohol ****in the past year****?

( ) Never

( ) Monthly or less

( ) 2 to 4 times a month

( ) 2 to 3 times a week

( ) 4 to 5 times a week

( ) 6 or more times a week

#### How many drinks did you have on a typical day when you were drinking ****in the past year****?

( ) 1 to 2 drinks

( ) 3 to 4 drinks

( ) 5 to 6 drinks

( ) 7 to 9 drinks

( ) 10 or more drinks

#### How often did you have 6 or more drinks on one occasion ****in the past year****?

( ) Never

( ) Less than monthly

( ) Monthly

( ) Weekly

( ) Daily or almost daily

## Substance Use: Injection Drug Use

### The next questions are about injection drug use. This means injecting drugs yourself or having someone who isn't a health care provider inject you.

#### Have you ****ever**** in your life shot up or injected any drugs other than those prescribed for you? By shooting up, we mean anytime you might have used drugs with a needle, either by mainlining, skin popping, or muscling.

( ) No

( ) Yes

( ) I prefer not to answer

( ) Don't know

#### ****In the past 12 months****, on average, how often did you inject?

( ) More than once a day

( ) Once a day

( ) More than once a week

( ) Once a week

( ) More than once a month

( ) Once a month

( ) Less than once a month

( ) Never

( ) I prefer not to answer

( ) Don't know

#### Which drug do you inject ****most often****?

( ) Speedball - Heroin and cocaine together

( ) Heroin, by itself

( ) Cocaine, by itself

( ) Crack

( ) Crystal, meth, tina, crank, ice

( ) Something else (Specify): _________________________________________________

( ) I prefer not to answer

( ) Don't know

## Substance Use: Non-Injection Drug Use

#### ****In the past 12 months****, have you used any non-injection drugs (drugs you did ****not**** inject), other than those prescribed for you.

( ) No

( ) Yes

( ) I prefer not to answer

( ) Don't know

#### ****In the past 12 months****, which drugs that were not prescribed to you did you use? (Check all that apply.)

[ ] Marijuana

[ ] Powdered cocaine (smoked or snorted)

[ ] Poppers (amyl nitrate)

[ ] X or Ecstasy

[ ] Painkillers (Oxycontin, Vicodin, Percocet)

[ ] Downers (Valium, Ativan, Xanax)

[ ] Crystal meth (tina, crank, ice)

[ ] Hallucinogens (LSD, mushrooms)

[ ] Special K (ketamine)

[ ] GHB

[ ] Crack cocaine

[ ] Other drug: _________________________________

[ ] Heroin (smoked or snorted)

[ ] I prefer not to answer

[ ] Don't know

## Substance Use: Drug use frequency

#### ****In the past 12 months****, how often did you use [DRUG NAME]?

( ) More than once a day

( ) Once a day

( ) More than once a week

( ) Once a week

( ) More than once a month

( ) Once a month

( ) Less than once a month

( ) I prefer not to answer

( ) Don't know

## Substance Use: Legal Marijuana

#### ****In the past 12 months****, have you been prescribed marijuana and had it filled at a legal dispensary?

( ) No

( ) Yes

( ) I prefer not to answer

( ) Don't know

## Sexual Behavior: Female Sex Partners

#### ****In the past 12 months**** (since in [MONTH/YEAR]), what types of sex have you had with a woman? (Check all that apply.)

#### By "having sex" we mean oral, vaginal, or anal sex. Oral sex means mouth on the vagina or penis; vaginal sex means penis in the vagina; and anal sex means penis in the anus (butt).

[ ] Oral sex

[ ] Vaginal sex

[ ] Anal sex

[ ] Some other type of sex

[ ] I have not had any type of sex with a woman in the past 12 months

[ ] I prefer not to answer

[ ] Don't know

## Sexual Behavior: Male Sex Partners, 2

### The next questions are about having sex with men. Oral sex means he put his mouth on your penis or you put your mouth on his penis. Anal sex means you put your penis in his anus (butt) or he put his penis in your anus (butt). Rimming means he put his mouth or tongue on your anus (butt) or you put your mouth or tongue on his anus (butt).

#### ****In the past 12 months**** (since in [MONTH/YEAR]), what types of sex have you had with other men?

[ ] Oral sex

[ ] Anal sex

[ ] Rimming

[ ] Some other type of sex

[ ] I have not had any type of sex with a man in the past 12 months

[ ] I prefer not to answer

[ ] Don't know

### Please specify other type of sex:

### _______________________________________________

## Male Sex Partners: Positioning

### The next questions are about having sex with men. Oral sex means he put his mouth on your penis or you put your mouth on his penis. Anal sex means you put your penis in his anus (butt) or he put his penis in your anus (butt). Rimming means he put his mouth or tongue on your anus (butt) or you put your mouth or tongue on his anus (butt).

## Now we will ask you to give some more detail about the sex you had with other men in the past 12 months. Please choose all statements that apply to you.

**In the past 12 months (since in [MONTH/YEAR]):** **I had anal sex as a top, i.e. I put my penis in someone else’s anus (butt)**

[ ] I had anal sex as a bottom, i.e. someone put his penis in my anus (butt)

[ ] I performed oral sex on someone

[ ] Someone performed oral sex on me

[ ] I rimmed someone

[ ] Someone rimmed me

[ ] I prefer not to answer

[ ] Don't know

## Sex partner number

### ****In the past 12 months**** (since in [MONTH/YEAR]), with how many different men have you had oral ****or**** anal sex?

_________________________________________________

### ****In the past 12 months****(since in [MONTH/YEAR]), with how many different men have you had ****anal**** sex?

_________________________________________________

### ****In the**** ****past 12 months****, since in [MONTH/YEAR], with how many different men have you had ****oral**** sex?

_________________________________________________

## Sexual Behavior: Male Sex Partners, 3

### Of the [NUMBER OF] men you had oral or anal sex with ****in the past 12 months****(since [MONTH/YEAR]), how many of them did you have ****anal**** sex with?

_________________________________________________

## Sexual Behavior: Condom Use

#### ****In the past 12 months****(since in [MONTH/YEAR]), did you have anal sex ****without using a condom****?

( ) No

( ) Yes

( ) I prefer not to answer

( ) Don't know

## Sexual Behavior: Condom Use, 2

### ****In the past 12 months****(since in [MONTH/YEAR]), with how many of these [NUMBER OF ANAL SEX PARTNERS] male anal sex partners did you have anal sex ****without using a condom****?

_________________________________________________

### ****In the past 12 months****(since [MONTH/YEAR]), with how many of these [NUMBER OF ANAL SEX PARTNERS] male anal sex partners did you have anal sex ****without using a condom****?

_________________________________________________

## Sexual Behavior: Male Sex Partners (1 Partner)

#### You mentioned that in the past 12 months, you had sex with one male partner.

#### ****In the past 12 months**** (since [MONTH/YEAR]), this male partner was a:

( ) Main partner (someone you felt committed to above anyone else)

( ) Casual partner (someone you didn't feel committed to or don't know very well)

( ) I prefer not to answer

( ) Don't know

#### As far as you know, during the time you were having a sexual relationship with your sexual partner, did ****he**** have sex with other people? Would you say he:

( ) Definitely did not

( ) Probably did not

( ) Probably did

( ) Definitely did

( ) I prefer not to answer

( ) Don't know

#### Did you know his HIV status?

( ) No

( ) Yes

( ) I prefer not to answer

#### What was his HIV status?

( ) HIV-negative

( ) HIV-positive

( ) Indeterminate

( ) I prefer not to answer

#### As far as you know, was he taking pre-exposure prophylaxis (PrEP or Truvada) to prevent HIV infection?

( ) No

( ) Yes

( ) I prefer not to answer

( ) Don't know

#### As far as you know, was he taking HIV medications (antiretrovirals) to treat his HIV infection? Some men will say that they are “undetectable” when taking HIV medications.

( ) No

( ) Yes

( ) I prefer not to answer

( ) Don't know

## Sexual Behavior: Male Sex Partners (>1)

#### ****In the past 12 months****(since [MONTH/YEAR]), the [TOTAL NUMBER SEX PARTNERS] male partners you told us about were:

( ) Only main partners (you felt committed to above anyone else)

( ) Only casual partners (you didn't feel committed to or don't know very well)

( ) Both main and casual partners

( ) I prefer not to answer

( ) Don't know

#### ****In the past 12 months**** (since [MONTH/YEAR]), did you have anal sex ****without using a condom**** with a man whose ****HIV status you did not know****?

( ) No

( ) Yes

( ) I prefer not to answer

( ) Don't know

#### Was this with a main or casual partner?

( ) Main partner

( ) Casual partner

( ) Both main and casual partners

( ) I prefer not to answer

( ) Don't know

#### ****In the past 12 months**** (since [MONTH/YEAR]) , did you have anal sex ****without using a condom**** with a man who was ****HIV positive****?

( ) No

( ) Yes

( ) I prefer not to answer

( ) Don't know

#### Was this with a main or casual partner?

( ) Main partner

( ) Casual partner

( ) Both main and casual partners

( ) I prefer not to answer

( ) Don't know

#### As far as you know, were these partners taking HIV medications (antiretrovirals) to treat their HIV-infection? Some men will say that they are “undetectable” when taking HIV medications.

( ) No

( ) Yes

( ) Some yes, some no

( ) I prefer not to answer

( ) Don't know

#### ****In the past 12 months****(since [MONTH/YEAR]), did you have anal sex ****without using a condom**** with a man who was ****HIV negative****?

( ) No

( ) Yes

( ) I prefer not to answer

( ) Don't know

#### Was this with a main or casual partner?

( ) Main partner

( ) Casual partner

( ) Both main and casual partners

( ) I prefer not to answer

( ) Don't know

#### As far as you know, were these partners taking pre-exposure prophylaxis (PrEP or Truvada) to prevent HIV infection?

( ) No

( ) Yes

( ) Some yes, some no

( ) I prefer not to answer

( ) Don't know

## Group Sex

### In the past 12 months, how many times did you have sex with ****more than one man in the same**** ****encounter**** (threesome or group sex)?

_________________________________________________

## Group Sex Numbers

### In the past 12 months, how many times did you have a ****threesome****, that is sex with two other men in the same encounter?

_________________________________________________

### In the past 12 months, how many times did you have ****group sex****, that is sex with three or more other men in the same encounter?

_________________________________________________

## Group Sex Without Condoms

#### The ****last time**** you had a threesome, with how many men did you have anal sex without a condom?

#### ( ) None (0)

#### ( ) 1 man

#### ( ) 2 men

### The last time you had group sex with three or more other men in the same encounter, with how many men did you have anal sex without a condom?

_________________________________________________

## Sexual Behavior: Social Habits

#### ****In the**** ****past 12 months****, have you exchanged things like money or drugs for sex with a male partner? Check all that apply.

[ ] No

[ ] Yes, I gave a sex partner things like drugs or money for sex

[ ] Yes, a sex partner gave me things like drugs or money for sex

[ ] I prefer not to answer

[ ] Don't know

#### ****In the past 12 months****, have you used any of the following kinds of internet sites to meet or socialize with gay men? Check all that apply.

[ ] Social network websites (such as Facebook)

[ ] Dating websites directed towards gay men

[ ] Mobile phone apps (such as gay chat, dating and hookup apps)

[ ] None of the above

[ ] I prefer not to answer

[ ] Don't know

#### How satisfied are you with your current sex life?

( ) Very satisfied

( ) Satisfied

( ) Unsure

( ) Dissatisfied

( ) Very Dissatisfied

( ) I prefer not to answer

( ) Don't know

## HIV Testing

#### Have you ****ever**** been tested for HIV? An HIV test checks whether someone has the virus that causes AIDS.

( ) No

( ) Yes

( ) I prefer not to answer

( ) Don't know

### ****In the past 2 years**** (since [MONTH/YEAR] of 2015), how many times have you been tested for HIV?

_________________________________________________

### When did you have your ****most recent**** HIV test? If you don't know the exact month, please enter your best guess.

#### Month:

( ) January

( ) February

( ) March

( ) April

( ) May

( ) June

( ) July

( ) August

( ) September

( ) October

( ) November

( ) December

Year: __________

## HIV Testing

#### Have you had an HIV test ****in the past 12 months**** (since in [MONTH/YEAR])?

( ) No

( ) Yes

( ) I prefer not to answer

( ) Don't know

#### When you most recently got tested in [MONTH/YEAR], where did you get tested?

( ) Private doctor's office

( ) HIV counseling and testing site

( ) Public health clinic/community health clinic

( ) Street outreach program/mobile unit

( ) Sexually transmitted disease clinic

( ) Hospital (inpatient)

( ) Correctional facility (jail or prison)

( ) Emergency room

( ) At home

( ) Other

( ) I prefer not to answer

( ) Don't know

## HIV Status

#### What was the result of your ****most recent**** HIV test in [MONTH/YEAR]?

( ) Negative

( ) Positive

( ) Never obtained results

( ) Indeterminate

( ) I prefer not to answer

( ) Don't know

#### Before your most recent test in [MONTH/YEAR], did you ****ever**** test positive for HIV?

( ) No

( ) Yes

( ) I prefer not to answer

( ) Don't know

#### Was your most recent test in [MONTH/YEAR] your ****first**** positive test?

( ) No

( ) Yes

( ) I prefer not to answer

( ) Don't know

### When did you ****first**** test positive?

#### Month

( ) January

( ) February

( ) March

( ) April

( ) May

( ) June

( ) July

( ) August

( ) September

( ) October

( ) November

( ) December

Year: _________________________________________________

## HIV Positive

#### Are you currently taking antiretroviral medicines to treat your HIV infection?

( ) No

( ) Yes

( ) I prefer not to answer

( ) Don't know

#### What is the ****main**** reason you are not currently taking any antiretroviral medicines?

( ) Not currently going to a health care provider for my HIV

( ) CD4 count and viral load are good

( ) Don't have money or insurance for antiretroviral medicines

( ) Don't want to take antiretroviral medicines

( ) Other

( ) I prefer not to answer

( ) Don't know

## HIV Positive: Non-US

#### Did you receive your first positive HIV test before or after you arrived in the United States to live?

( ) No

( ) Yes

( ) I prefer not to answer

( ) Don't know

#### Were you taking HIV medicines before arriving in the United States to live?

( ) No

( ) Yes

( ) I prefer not to answer

( ) Don't know

#### Have you experienced any of the following barriers to HIV care or taking HIV medicines in the U.S.? Check all that apply.

[ ] Health care provider doesn’t speak my language

[ ] Pharmacist doesn’t speak my language

[ ] I didn’t have access to an interpreter

[ ] I was worried that an interpreter might disclose my HIV status or sexual orientation to others in my community

[ ] I was worried that a health care provider would share my information with law or immigration enforcement

[ ] I was worried that a health care provider would discriminate against me because of my sexual orientation or gender identity

[ ] I was worried that a health care provider would discriminate against me because of my race/ethnicity

[ ] I was worried that a health care provider would discriminate against me because of my country of origin

[ ] Health care provider doesn’t understand my culture

[ ] I don’t have insurance

[ ] None of the above

[ ] I prefer not to answer

[ ] Don't know

## PrEP Module

#### ****Before today****, have you ****ever**** heard of people who do not have HIV taking PrEP, the antiretroviral medicine taken every day for months or years to reduce the risk of getting HIV?

( ) No

( ) Yes

## PrEP Use

#### ****In the past 12 months****(since [MONTH/YEAR]), have you had a discussion with a health care provider about taking PrEP?

( ) No

( ) Yes

( ) I prefer not to answer

( ) Don't know

#### ****In the past 12 months****(since [MONTH/YEAR]), have you taken PrEP?

( ) No

( ) Yes

( ) I prefer not to answer

( ) Don't know

#### ****H****ave you ever taken PrEP?

( ) No

( ) Yes

( ) I prefer not to answer

( ) Don't know

#### Which of the following people or places describe how you got PrEP during the last 12 months? Check all that apply.

[ ] Prescription from a healthcare provider

[ ] Directly from a healthcare provider or clinic

[ ] A friend or relative

[ ] A person you had/have sex with

[ ] Health department

[ ] Online

[ ] Some other source

[ ] I prefer not to answer

[ ] Don't know

### What was that other source for PrEP?

_________________________________________________

## PrEP discussions with HCP

#### When first discussing PrEP with your healthcare provider, who started the conversation?

( ) I asked my healthcare provider about PrEP

( ) My healthcare provider told me about PrEP

( ) I prefer not to answer

( ) Don't know

#### After discussing PrEP with a healthcare provider, have you ever asked to start PrEP but been denied?

( ) Yes

( ) No

( ) I decided not to start PrEP after discussing it with my provider

( ) I prefer not to answer

( ) Don't know

#### What was the primary reason your healthcare provider told you for not prescribing PrEP?

( ) He/She did not know how to prescribe PrEP

( ) I was not at high risk for HIV

( ) I might already have HIV and needed further testing

( ) I should not be on PrEP because of my medical condition

( ) I would not be able to take a daily pill and follow up regularly

( ) I would not be able to afford PrEP

( ) PrEP was not effective for preventing HIV

( ) PrEP would encourage unsafe sex

( ) Some other reason

( ) No reason given or unsure

### What was the other reason your provider gave for not prescribing you PrEP?

_________________________________________________

#### By not being prescribed PrEP, did you feel discriminated against based on any of the following? Check all that apply.

[ ] Race/ethnicity

[ ] Sexual orientation

[ ] Gender identity

[ ] Economic status

[ ] Other (please specify):

[ ] I did not experience discrimination

## Current PrEP Use

#### Are you currently taking PrEP?

( ) No

( ) Yes

( ) I prefer not to answer

( ) Don't know

#### ****In the last 30 days****, about how many doses of PrEP did you take?

( ) Less than 15

( ) 16-29

( ) 30

#### How many months in a row have you been taking PrEP?

( ) Less than 2 months

( ) 2 to 6 months

( ) 7 to 12 months

( ) 12 months or more

( ) I prefer not to answer

( ) Don't know

#### Now that you are taking PrEP, how has your condom use changed?

( ) I use condoms less of the time

( ) I use condoms the same amount of the time

( ) I use condoms more of the time

( ) I prefer not to answer

( ) Don't know

## PrEP use discontinued

#### The ****last time**** you were on PrEP, how many months in a row were you taking it?

( ) Less than 2 months

( ) 2 to 6 months

( ) 7 to 12 months

( ) I prefer not to answer

( ) Don't know

#### The last time you were on PrEP, ****about how many doses of PrEP per month**** did you typically take?*

( ) Less than 15

( ) 16-29

( ) 30

#### Which of the following describes the reason(s) why you stopped using PrEP the last time you were on it: (Check all that apply)

[  ] The cost was too high

[  ] I lost my job and/or insurance

[  ] I was concerned about the side-effects

[  ] I could not remember to take the pill every day

[  ] I started a monogamous relationship with an HIV-negative partner

[  ] I prefer to use other methods to protect myself from HIV

[  ] I was worried that people will think that I have HIV when they see me taking the pill

[  ] I was worried that people will know that I have sex with men or transgender people

[  ] I was worried people will think I very sexually active because I am on PrEP

[  ] Other reason, please specify:

## PrEP willingness

#### Would you be willing to take anti-HIV medicines ****every day**** to lower your chances of getting HIV?

( ) No

( ) Yes

( ) I prefer not to answer

( ) Don't know

#### What might make you consider starting take anti-HIV medicines every day to lower your chances of getting HIV? Check all that apply.

[ ] Recommendation from my healthcare provider

[ ] Recommendation from a friend

[ ] Change in my relationship status/sexual behavior

[ ] Advertisements

[ ] Additional information about PrEP

[ ] Nothing

[ ] I prefer not to answer

[ ] Don’t know

#### Why would you not be willing to take anti-HIV medicines every day to lower your chances of getting HIV?

_________________________________________________

## PEP

#### ****In the past 12 months****(since [MONTH/YEAR]), have you taken PEP to reduce the risk of getting HIV?

( ) No

( ) Yes

( ) Don't know

( ) I prefer not to answer

#### Did your PEP provider mention PrEP as an option for you to reduce your risk of HIV in the future?

( ) No

( ) Yes

( ) Don't know

( ) I prefer not to answer

#### In the United States, have you experienced any of the following barriers to getting HIV prevention services, such as HIV testing, PrEP or PEP? Check all that apply.

[ ] Health care provider doesn’t speak my language

[ ] Pharmacist doesn’t speak my language

[ ] I didn’t have access to an interpreter

[ ] I was worried that an interpreter might disclose my HIV status or sexual orientation to others in my community

[ ] I was worried that a health care provider would share my information with law or immigration enforcement

[ ] I was worried that a health care provider would discriminate against me because of my sexual orientation or gender identity

[ ] I was worried that a health care provider would discriminate against me because of my race/ethnicity

[ ] I was worried that a health care provider would discriminate against me because of my country of origin

[ ] Health care provider doesn’t understand my culture

[ ] I don’t have insurance

[ ] None of the above

[ ] I prefer not to answer

[ ] Don't know

## Social Cohesion

### Please indicate how much you agree or disagree with the following statements.

| I feel that I belong and am a part of my neighborhood. | **Strongly Agree** | **Agree** | **Disagree** | **Strongly Disagree** | **Prefer not to answer** | **Don’t know** |
| --- | --- | --- | --- | --- | --- | --- |
| People around here are willing to help their neighbors. | **Strongly Agree** | **Agree** | **Disagree** | **Strongly Disagree** | **Prefer not to answer** | **Don’t know** |
| This is a close-knit neighborhood. | **Strongly Agree** | **Agree** | **Disagree** | **Strongly Disagree** | **Prefer not to answer** | **Don’t know** |
| People in this neighborhood can be trusted. | **Strongly Agree** | **Agree** | **Disagree** | **Strongly Disagree** | **Prefer not to answer** | **Don’t know** |
| People in this neighborhood generally don't get along with each other. | **Strongly Agree** | **Agree** | **Disagree** | **Strongly Disagree** | **Prefer not to answer** | **Don’t know** |
| People in this neighborhood do not share the same values. | **Strongly Agree** | **Agree** | **Disagree** | **Strongly Disagree** | **Prefer not to answer** | **Don’t know** |

## Dating site features

#### Do you know that you can sign up for testing reminders through some of the dating and hook-up apps? The feature sends a message to your in-box at a 3- or 6- month interval that you choose and enables you to find a testing site near you.

( ) No

( ) Yes

( ) I prefer not to answer

( ) Don't know

#### Did you opt into using the testing reminder feature?

( ) No

( ) Yes

( ) I prefer not to answer

( ) Don't know

#### Is the testing reminder feature one you would like to use in the future?

( ) No

( ) Yes

( ) I prefer not to answer

( ) Don't know

## Dating site features

#### Do you know that on many apps you can include your preferred sexual health strategy, like condoms, PrEP, or being on treatment for HIV?

( ) No

( ) Yes

( ) I prefer not to answer

( ) Don't know

#### Did you opt into using the preferred sexual health strategy feature?

( ) No

( ) Yes

( ) I prefer not to answer

( ) Don't know

#### Which options did you select? Check all that apply.

[ ] Condoms

[ ] PrEP

[ ] Treatment as Prevention / Taking HIV meds / Undetectable

#### Is the preferred sexual health strategy feature one you would like to use in the future?

( ) No

( ) Yes

( ) I prefer not to answer

( ) Don't know

## Dating site features

#### To what extent, if at all, are you are you stressed or bothered by how people treat each other when using dating apps?

( ) Extremely stressed/bothered

( ) Very stressed/bothered

( ) Moderately stressed/bothered

( ) Slightly stressed/bothered

( ) Not at all stressed/bothered

#### Have you ever experienced any of the following on dating sites or apps? Check all that apply.

[ ] Ghosting

[ ] Rejection

[ ] Rude comments

[ ] Discrimination

[ ] None of above

[ ] I prefer not to answer

[ ] Don't know

## Stigma - NHBS Measure

#### ****In the past 12 months****, have any of the following things happened to you because someone knew or assumed you were attracted to men?

|  | **Yes** | **No** | **I prefer not to answer** | **Don't know** | **Does not apply** |
| --- | --- | --- | --- | --- | --- |
| You were called names or insulted | ( ) | ( ) | ( ) | ( ) | ( ) |
| You received poorer services than other people in restaurants, stores, other businesses or agencies | ( ) | ( ) | ( ) | ( ) | ( ) |
| You were treated unfairly at work or school | ( ) | ( ) | ( ) | ( ) | ( ) |
| You were denied or given lower quality health care | ( ) | ( ) | ( ) | ( ) | ( ) |
| You were physically attacked or injured | ( ) | ( ) | ( ) | ( ) | ( ) |

#### How much do you agree or disagree with the following: Most people in my area are tolerant of gays and bisexuals.

( ) Strongly agree

( ) Agree

( ) Neither agree or disagree

( ) Disagree

( ) Strongly disagree

( ) I prefer not to answer

( ) Don't know

## Stigma

#### Have you ever felt excluded from family activities because you have sex with men?

( ) No

( ) Yes, in the last 6 months

( ) Yes, but not in the last 6 months

( ) I prefer not to answer

( ) Don’t know

#### Have you ever felt that family members have made discriminatory remarks or gossiped about you because you have sex with men?

( ) No

( ) Yes, in the last 6 months

( ) Yes, but not in the last 6 months

( ) I prefer not to answer

( ) Don’t know

#### Have you ever felt rejected by your friends because you have sex with men?

( ) No

( ) Yes, in the last 6 months

( ) Yes, but not in the last 6 months

( ) I prefer not to answer

( ) Don’t know

## Stigma

#### Have you ever felt afraid to go to health care services because you worry someone may learn you have sex with men?

( ) No

( ) Yes, in the last 6 months

( ) Yes, but not in the last 6 months

( ) I prefer not to answer

( ) Don’t know

#### Have you ever avoided going to health care services because you worry someone may learn you have sex with men?

( ) No

( ) Yes, in the last 6 months

( ) Yes, but not in the last 6 months

( ) I prefer not to answer

( ) Don’t know

#### Have you ever heard health care providers gossiping about you (talking about you) because you have sex with men?

( ) No

( ) Yes, in the last 6 months

( ) Yes, but not in the last 6 months

( ) I prefer not to answer

( ) Don’t know

#### Have you ever felt that you were not treated well in a health center because someone knew that you have sex with men?

( ) No

( ) Yes, in the last 6 months

( ) Yes, but not in the last 6 months

( ) I prefer not to answer

( ) Don’t know

## Stigma

#### Have you ever felt that the police refused to protect you because you have sex with men?

( ) No

( ) Yes, in the last 6 months

( ) Yes, but not in the last 6 months

( ) I prefer not to answer

( ) Don’t know

#### Have you ever felt scared to be in public places because you have sex with men?

( ) No

( ) Yes, in the last 6 months

( ) Yes, but not in the last 6 months

( ) I prefer not to answer

( ) Don’t know

#### Have you ever been verbally harassed and felt it was because you have sex with men?

( ) No

( ) Yes, in the last 6 months

( ) Yes, but not in the last 6 months

( ) I prefer not to answer

( ) Don’t know

#### Have you ever been blackmailed by someone because you have sex with men?

( ) No

( ) Yes, in the last 6 months

( ) Yes, but not in the last 6 months

( ) I prefer not to answer

( ) Don’t know

## Stigma

#### Has someone ever physically hurt you (pushed, shoved, slapped, hit, kicked, choked or otherwise physically hurt you)?

( ) No

( ) Yes, in the last 6 months

( ) Yes, but not in the last 6 months

( ) I prefer not to answer

( ) Don’t know

#### Do you believe any of these experiences of physical violence was/were related to the fact that you have sex with men?

( ) No

( ) Yes, in the last 6 months

( ) Yes, but not in the last 6 months

( ) I prefer not to answer

( ) Don’t know

#### Have you ever been forced to have sex when you did not want to? By forced, we mean physically forced, coerced to have sex, or penetrated with an object, when you did not want to.

( ) No

( ) Yes, in the last 6 months

( ) Yes, but not in the last 6 months

( ) I prefer not to answer

( ) Don’t know

#### Do you believe any of these experiences of sexual violence were related to the fact that you have sex with men?

( ) No

( ) Yes, in the last 6 months

( ) Yes, but not in the last 6 months

( ) I prefer not to answer

( ) Don’t know

## K-6 Mental Health Scale

#### During the past ****30 days****, about how often did you feel...

|  | **All of the time** | **Most of the time** | **Some of the time** | **A little of the time** | **None of the time** | **I prefer not to answer** | **Don't know** |
| --- | --- | --- | --- | --- | --- | --- | --- |
| **{K6_NERVOUS}** Nervous | ( ) | ( ) | ( ) | ( ) | ( ) | ( ) | ( ) |
| **{K6_HOPELESS}**  Hopeless | ( ) | ( ) | ( ) | ( ) | ( ) | ( ) | ( ) |
| **{K6_RESTLESS}**  Restless or fidgety | ( ) | ( ) | ( ) | ( ) | ( ) | ( ) | ( ) |
| **{K6_DEPRESSED}**  So depressed that nothing could cheer you up | ( ) | ( ) | ( ) | ( ) | ( ) | ( ) | ( ) |
| **{K6_EFFORT}**  That everything was an effort | ( ) | ( ) | ( ) | ( ) | ( ) | ( ) | ( ) |
| **{K6_WORTHLESS}**Worthless | ( ) | ( ) | ( ) | ( ) | ( ) | ( ) | ( ) |

## Suicidality

#### At any time in the past 12 months, up to and including today, did you seriously think about trying to kill yourself?

( ) No

( ) Yes

( ) I prefer not to answer

( ) Don't know

#### During the past 12 months, did you make any plans to kill yourself?

( ) No

( ) Yes

( ) I prefer not to answer

( ) Don't know

#### During the past 12 months, did you try to kill yourself?

( ) No

( ) Yes

( ) I prefer not to answer

( ) Don't know

## STD Testing

**Have you ever been tested for the sexually transmitted infections gonorrhea, chlamydia, or syphilis?**

( ) No

( ) Yes

( ) I prefer not to answer

( ) Don't know

**In the past 12 months, that is, since [MONTH/YEAR], were you tested by a doctor or other health care provider for a sexually transmitted infection like gonorrhea, chlamydia, or syphilis?**

( ) No

( ) Yes

( ) I prefer not to answer

( ) Don't know

**In the past 12 months, when you were tested by a doctor or other health care provider for a sexually transmitted infection like gonorrhea, chlamydia, or syphilis, what samples did you provide for testing? Check all that apply.**

[ ] I had my blood drawn

[ ] I gave a urine sample

[ ] I had my rectum (butt) swabbed

[ ] I had my throat swabbed

[ ] I prefer not to answer

[ ] Don't know

## Bacterial STI Diagnoses

#### ****In the past 12 months**** (since in [MONTH/YEAR]), has a doctor, nurse or other health care provider told you that you had any of the following? Check all that apply.

[ ] Gonorrhea

[ ] Chlamydia

[ ] Syphilis

[ ] None of the above

[ ] I prefer not to answer

[ ] Don't know

**Electronic Personal Health Records**

#### Are you able to view your health records online, for example, with MyChart or another electronic personal health record (PHR) system?

#### ( ) No

#### ( ) Yes

#### ( ) I prefer not to answer

#### ( ) Don’t know

#### Do you want the option to securely view your online health records?

#### **( ) No**

#### **( ) Yes**

#### **( ) I prefer not to answer**

#### **( ) Don’t know**

#### What is your opinion about online access to sexually transmitted infection (STI) test results - like gonorrhea or syphilis?

|  | **Strongly Agree** | **Agree** | **Disagree** | **Strongly Disagree** | **I prefer not to answer** | **Don't know** |
| --- | --- | --- | --- | --- | --- | --- |
| **{PHR_CONVEN}** It is a more convenient way to manage my sexual health records. | ( ) | ( ) | ( ) | ( ) | ( ) | ( ) |
| **{PHR_AWARE}**  It encourages people to be more aware of their sexual health. | ( ) | ( ) | ( ) | ( ) | ( ) | ( ) |
| **{PHR_DECISON}**  It will help people like me make better sexual health decisions. | ( ) | ( ) | ( ) | ( ) | ( ) | ( ) |

#### What type of device do you or would you use to access your online health record? Choose ****all that apply.****

[ ] Computer

[ ] Laptop

[ ] iPad or other type of electronic tablet

[ ] Phone

[ ] I would not access my STI test results electronically or online

[ ] Other (please specify): _____________

#### With online access to your HIV and STD test results, how likely would you share it with a ****main**** partner? By main partner, we mean someone you feel committed to above anyone else.

#### ( ) Very unlikely

#### ( ) Unlikely

#### ( ) Likely

#### ( ) Very likely

#### ( ) I prefer not to answer

#### ( ) Don’t know

#### With online access to your HIV and STD test results, how likely would you share it with a ****casual**** partner? By casual partner, we mean someone you don't feel committed to or don't know very well.

#### ( ) Very unlikely

#### ( ) Unlikely

#### ( ) Likely

#### ( ) Very likely

#### ( ) I prefer not to answer

#### ( ) Don’t know

#### ****Please indicate to what extent you believe exchanging online HIV and STD test results between you and your sexual partners will:****

|  | **Definitely not** | **Probably not** | **Probably** | **Definitely** | **I prefer not to answer** | **Don't know** |
| --- | --- | --- | --- | --- | --- | --- |
| **{PHR_IMPROVE1}** Improve communication on HIV and other sexually transmitted infections? | ( ) | ( ) | ( ) | ( ) | ( ) | ( ) |
| **{PHR_IMPROVE2}**  Improve my confidence in the testing information a partner shares with me? | ( ) | ( ) | ( ) | ( ) | ( ) | ( ) |
| **{PHR_IMPROVE3}**  Improve control over my sexual health and decision making? | ( ) | ( ) | ( ) | ( ) | ( ) | ( ) |

#### ****In your opinion, rank the value of including the following services as electronic health record features:****

|  | **High Value** | **Moderate Value** | **Low Value** | **No Value** | **I prefer not to answer** | **Don't know** |
| --- | --- | --- | --- | --- | --- | --- |
| Tips/tools for managing sexual health | ( ) | ( ) | ( ) | ( ) | ( ) | ( ) |
| Ability to locate STD test centers and services | ( ) | ( ) | ( ) | ( ) | ( ) | ( ) |
| Video chat for communicating with healthcare providers | ( ) | ( ) | ( ) | ( ) | ( ) | ( ) |
| Counseling and resources for people with STDs | ( ) | ( ) | ( ) | ( ) | ( ) | ( ) |
| Ability of order home test kits for HIV and STDs | ( ) | ( ) | ( ) | ( ) | ( ) | ( ) |
| Games to promote sexual health | ( ) | ( ) | ( ) | ( ) | ( ) | ( ) |
| Other | ( ) | ( ) | ( ) | ( ) | ( ) | ( ) |

### Please suggest another electronic health record feature:

_________________________________________________

## Assessment of Prevention Activities

#### ****In the**** ****past 12 months****, have you gotten any free condoms, not counting those given to you by a friend, relative, or sex partner?

( ) No

( ) Yes

( ) I prefer not to answer

( ) Don't know

#### ****In the past 12 months****, have you had a one-on-one conversation with an outreach worker, counselor, or prevention program worker about ways to prevent HIV? Don't count the times where you had a conversation as part of an HIV test.

( ) No

( ) Yes

( ) I prefer not to answer

( ) Don't know

#### ****In the**** ****past 12 months****, have you been a participant in any organized session(s) involving a small group of people to discuss ways to prevent HIV? Don't include discussions you had with a group of friends.

( ) No

( ) Yes

( ) I prefer not to know

( ) Don't know

## U=U

#### Do you know what it means if a person living with HIV is called "undetectable"?

( ) No

( ) Yes

( ) I prefer not to know

( ) Don't know

#### Have you heard of the slogan “Undetectable=Untransmittable” (U=U)?

( ) No

( ) Yes

( ) I prefer not to know

( ) Don't know

#### How did you hear about “Undetectable=Untransmittable” (U=U)? (select all that apply)

#### [ ] Online media (article)

#### [ ] Print media (ads, magazines)

#### [ ] Social media (e.g. Twitter, Facebook)

#### [ ] Dating/hookup app or site (e.g. Scruff)

#### [ ] Word of mouth (e.g. from a friend or acquaintance)

#### [ ] Other

#### [ ] I don’t know

### What is the other way you heard about "Undetectable=Untransmittable" (U=U)?

_________________________________________________

## U=U

#### With regard to HIV-positive individuals transmitting HIV through sexual contact, how accurate do you believe the slogan “Undetectable=Untransmittable” is?

#### ( ) Completely accurate

#### ( ) Somewhat accurate

#### ( ) Somewhat inaccurate

#### ( ) Completely inaccurate

#### ( ) I don’t know

#### If a person living with HIV has an undetectable viral load, how likely do you think they would be to transmit HIV to their partner if they have unprotected sex?

0 ________________________[__]_____________________________ 100

## U=U

#### For some people, knowing about U=U has led to changes. Which of the following has been your experience as a result of hearing about U=U?

#### ( ) I am more likely to discuss HIV status with sexual partners

#### ( ) I am less likely to discuss HIV status with sexual partners

#### ( ) I have made no changes in how often I discuss HIV status with sexual partners

#### ( ) Not applicable

#### ( ) I prefer not to answer

#### ( ) Don’t know

#### Which of the following has been your experience as a result of hearing about U=U? With partners of positive or unknown HIV status:

( ) I use condoms more

( ) I use condoms less

( ) My condom use has not changed

#### ( ) Not applicable

#### ( ) I prefer not to answer

#### ( ) Don’t know

## U=U

#### Do you know what it means if a person living with HIV is called "undetectable"?

#### ( ) I will be more likely to discuss HIV status with sexual partners

#### ( ) I will be less likely to discuss HIV status with sexual partners

#### ( ) I will make no changes in how often I discuss HIV status with sexual partners

#### ( ) Not applicable

#### ( ) I prefer not to answer

#### ( ) Don’t know

#### Which of the following do you think will happen as a result of hearing about U=U? With partners of positive or unknown HIV status:

( ) I will use condoms more

( ) I will use condoms less

( ) My condom use will not changed

#### ( ) Not applicable

#### ( ) I prefer not to answer

#### ( ) Don’t know

#### ( ) I don’t know

#### ( ) Don’t know

## Prevention Campaigns

### Thank you for staying with us! You are almost done the survey.

### ****In the past 12 months****, how often did you see or hear the following slogans or messages?

#### Let's stop HIV together:

( ) Never

( ) Rarely

( ) Sometimes

( ) Often

( ) Very Often

( ) I prefer not to answer

( ) Don't know

#### On a scale of 0 to 5, where 0 means "not very effective" and 5 means "very effective", how effective do you think this slogan or message is?

( ) 0 (Not Effective)

( ) 1

( ) 2

( ) 3

( ) 4

( ) 5 (Very Effective)

#### Protest:

( ) Never

( ) Rarely

( ) Sometimes

( ) Often

( ) Very Often

( ) I prefer not to answer

( ) Don't know

#### On a scale of 0 to 5, where 0 means "not very effective" and 5 means "very effective", how effective do you think this slogan or message is?

( ) 0 (Not Effective)

( ) 1

( ) 2

( ) 3

( ) 4

( ) 5 (Very Effective)


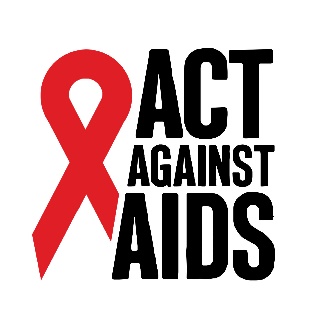


#### ACT Against AIDS:

( ) Never

( ) Rarely

( ) Sometimes

( ) Often

( ) Very Often

( ) I prefer not to answer

( ) Don't know

#### On a scale of 0 to 5, where 0 means "not very effective" and 5 means "very effective", how effective do you think this slogan or message is?

( ) 0 (Not Effective)

( ) 1

( ) 2

( ) 3

( ) 4

( ) 5 (Very Effective)

#### Greater than AIDS:

( ) Never

( ) Rarely

( ) Sometimes

( ) Often

( ) Very Often

( ) I prefer not to answer

( ) Don't know

#### On a scale of 0 to 5, where 0 means "not very effective" and 5 means "very effective", how effective do you think this slogan or message is?

( ) 0 (Not Effective)

( ) 1

( ) 2

( ) 3

( ) 4

( ) 5 (Very Effective)

#### Start Talking. Stop HIV.

( ) Never

( ) Rarely

( ) Sometimes

( ) Often

( ) Very Often

( ) I prefer not to answer

( ) Don't know

#### On a scale of 0 to 5, where 0 means "not very effective" and 5 means "very effective", how effective do you think this slogan or message is?

( ) 0 (Not Effective)

( ) 1

( ) 2

( ) 3

( ) 4

( ) 5 (Very Effective)

#### HIV Treatment Works:

( ) Never

( ) Rarely

( ) Sometimes

( ) Often

( ) Very Often

( ) I prefer not to answer

( ) Don't know

#### On a scale of 0 to 5, where 0 means "not very effective" and 5 means "very effective", how effective do you think this slogan or message is?

( ) 0 (Not Effective)

( ) 1

( ) 2

( ) 3

( ) 4

( ) 5 (Very Effective)

## Prevention Campaigns

### This is the last prevention message we will show you and it's a short video ad. The following video discusses sex and HIV prevention. If you are in a public space, you may want to reduce your volume, mute your device, or use headphones when viewing this ad.

#### In the past 12 months, did you see this ad for Truvada for PrEP from Gilead Sciences?

( ) Yes

( ) No

( ) Don't know

#### On a scale of 0 to 5, where 0 means "not very effective" and 5 means "very effective", how effective do you think this ad is?

( ) 0 (Not Effective)

( ) 1

( ) 2

( ) 3

( ) 4

( ) 5 (Very Effective)

## Study Target Inquiries

#### As far as you know, did you participate in the “Sex is the Question” Survey between July 2017 and November 2017?

( ) Yes

( ) No

( ) I'm not sure

### For this national study, we are recruiting a large number of men like you. Can you tell us the name of social networking website or app where we could reach other men like you who might like to complete this survey?

_________________________________________________

## Future Contact

#### The PRISM Health team conducts many research projects at Emory University. Would you like to be contacted for potential participation in our future projects?

( ) Yes

( ) No

### Please provide the email address you would like for us to use to contact you for future studies.

_________________________________________________
